# *Notes from the Field:* Responding to an Outbreak of Monkeypox Using the One Health Approach — Nigeria, 2017–2018

**DOI:** 10.15585/mmwr.mm6737a5

**Published:** 2018-09-21

**Authors:** Womi-Eteng Eteng, Anna Mandra, Jeff Doty, Adesola Yinka-Ogunleye, Sola Aruna, Mary G. Reynolds, Andrea M. McCollum, Whitni Davidson, Kimberly Wilkins, Muhammad Saleh, Oladipupo Ipadeola, Lamin Manneh, Uchenna Anebonam, Zainab Abdulkareem, Nma Okoli, Jeremiah Agenyi, Chioma Dan-Nwafor, Ibrahim Mahmodu, Chikwe Ihekweazu

**Affiliations:** ^1^Nigeria Centre for Disease Control, Abuja, Nigeria; ^2^Division of High-Consequence Pathogens and Pathology, National Center for Emerging and Zoonotic Infectious Diseases, CDC; ^3^Epidemic Intelligence Service, CDC; ^4^Measure Evaluation Nigeria, Nigeria; ^5^Divison of Global Health Protection, Center for Global Health, CDC; ^6^Nigeria Field Epidemiology and Laboratory Training Program, Abuja, Nigeria; ^7^Federal Ministry of Agriculture and Rural Development, Abuja, Nigeria; ^8^World Health Organization Country Office, Abuja, Nigeria.

On September 22, 2017, a suspected human case of monkeypox was reported to the Nigeria Centre for Disease Control (NCDC) from Bayelsa State in southern Nigeria. Because monkeypox had not been reported in Nigeria since 1978 (*1*), the case raised national and international concern. A multisectoral, international outbreak investigation was undertaken to identify sources and risk factors, establish surveillance, and enhance preparedness. A suspected case was defined as the sudden onset of fever, followed by a vesiculopustular rash primarily on the face, palms, and soles. A confirmed case was any suspected case with laboratory confirmation (by serology, molecular detection of viral DNA, or virus isolation). A probable case was a suspected case epidemiologically linked to a confirmed case. As of February 25, 2018, a total of 228 suspected cases (including 89 confirmed and three probable cases) had been investigated in 24 of Nigeria’s 36 states and the Federal Capital Territory. Six deaths (6.7%) were recorded among the 89 confirmed cases. The outbreak has not been declared over, and NCDC continues to collect data to develop a baseline level for this disease, which had not been reported in 40 years and now might be endemic to Nigeria. Given the zoonotic nature of the disease, this outbreak has required a robust One Health outbreak collaboration among human, animal, and environmental health institutions.

Monkeypox virus is a zoonotic orthopoxvirus. Although the animal reservoir is not known, small mammals appear to play a role in the circulation of the virus in nature (*2*). Monkeypox virus can be transmitted to humans through bites and direct contact with infected animals, including during preparation of meat, and case fatality rates can reach 10%. Currently no drugs are licensed for treatment of monkeypox; smallpox vaccine, which historically demonstrated approximately 85% protection against monkeypox, has not been in widespread use since the eradication of smallpox in 1980 (*3**,**4*).

A multiagency interdisciplinary emergency operations center (EOC) was activated on October 9, 2017; the EOC facilitated joint epidemiologic investigations, targeted risk communication, and developed laboratory diagnostic capacity for human and animal specimens.* An incident action plan and interim national guidelines were developed, and a protocol for active monkeypox surveillance in animals was developed, targeting high-risk areas at the human-animal interface, such as markets that sell bush meat (meat from nondomesticated animals hunted for food), wildlife parks, zoos, and farms. To enhance laboratory diagnostic capacity, personnel from the NCDC National Reference Laboratory and the National Veterinary Research Institute received training in monkeypox molecular diagnosis.

Joint human and animal health teams conducted field investigations to study the human, animal, and environmental sources of infection, as well as risk factors and modes of transmission. Human-to-human transmission was presumed in a limited number of cases through investigation into clusters (within individual households) of confirmed cases. A human-to-human transmission chain was presumed when symptom onset occurred in a close contact of a confirmed case at an interval consistent with the incubation period of 5–13 days. Most cases could not be epidemiologically linked, suggesting a multisource outbreak or previously undetected endemic transmission. Links to zoonotic origin also could not be determined, and the role of environmental factors is not known. Further institutional collaboration for research in these areas has been identified. The communications team developed and implemented a plan focused on alleviating public fear and anxiety regarding this largely unknown disease. Key messages, health advisories, frequently asked questions, press releases, and a risk communication activity tracker were formulated in collaboration with animal health partners with contents addressing possible risk factors identified during the investigation. Key messages included avoiding physical contact with persons infected with monkeypox, avoiding contact with wild animals (especially those found dead), cooking animal food products thoroughly before consumption, frequent handwashing, and early medical evaluation of persons with compatible signs or symptoms.

This outbreak likely resulted from a complex intersection of events and, given the zoonotic nature of the disease, required a robust outbreak response collaboration among human, animal, and environmental health institutions. The Economic Community of West African States, in partnership with its member states, has in the past adopted a One Health multidisciplinary approach to human, animal, and environmental health in implementing outbreak response and preparedness, surveillance, communications, coordination, and epidemiologic investigations (*5*). This method facilitates efficient use of scarce resources and leverages various sectors’ capabilities.

The response to this outbreak demonstrates the utility of multisectoral collaboration for the investigation and control of zoonotic disease outbreaks. As is best practice in emergency management models, an after-action review involving all partners will be critical in upholding successes, addressing weaknesses, and preparing for future outbreaks.

## Footnotes

* The EOC included representatives from the Federal Ministry of Agriculture and Rural Development; World Health Organization; CDC; African Field Epidemiology Network; University of Maryland; United National Children’s Fund; Africa CDC; and eHealth Africa.
